# Supplementary material for: Empirical methods for controlling false positives and estimating confidence in ChIP-Seq peaks
Source: BMC Bioinformatics. 2008 Dec 5;9:523. doi: 10.1186/1471-2105-9-523 (PMC2628906; doi:10.1186/1471-2105-9-523)
Supplement: Additional File 1 — A variety of html documents from the USeq web site detailing the available applications, their best usage, output file type descriptions, command line menus, etc. [file 1471-2105-9-523-S1.zip › USeqUserGuides/JavaDoc.zip!/JavaDoc/trans/misc/package-tree.html]

trans.misc Class Hierarchy


|  |  |  |
| --- | --- | --- |
| **Overview** \| **Package** \| Class \| Use \| **Tree** \| **Deprecated** \| **Index** \| **Help** | |  |
| **PREV**   **NEXT** | **FRAMES**    **NO FRAMES**     **All Classes** |  |


---


## Hierarchy For Package trans.misc

**Package Hierarchies:**: All Packages

---

## Class Hierarchy

- java.lang.Object
  - trans.misc.**AverageDoubles**- trans.misc.**Bar2Gr**- trans.misc.**Bed2Gr**- trans.misc.**BestWindowScoreExtractor**- trans.misc.**CalcGCAndTransform**- trans.misc.**CalcGCRatiosFromIntervals**- trans.misc.**CalculateGeneExtensions**- trans.misc.**ConvertAgilentData**- trans.misc.**ConvertBars2TPMapCel**- trans.misc.**ConvertGeoData**- trans.misc.**ConvertNimblegenNDF2TPMap**- trans.misc.**ConvertNimblegenPAIR2Cela**- trans.misc.**CorrelateGrFiles**- trans.misc.**Delme**- trans.misc.**GCRatio** (implements java.lang.Comparable<T>)- trans.misc.**Gr2Bar**- trans.misc.**GrGraph**- trans.misc.**InefficientPseudoMedian**- trans.misc.**PrependColumn**- trans.misc.**Regions2Intervals**- trans.misc.**ScoredRegionsFormatFixer**- trans.misc.**Sgr2Bar**- trans.misc.**SgrComparator** (implements java.util.Comparator<T>)- trans.misc.**TextPValOrSig2Gr**- trans.misc.**TransformRatios**- trans.misc.**Util**- trans.misc.**Wig2Bar**- trans.misc.**WindowHistogram**

---


|  |  |  |
| --- | --- | --- |
| **Overview** \| **Package** \| Class \| Use \| **Tree** \| **Deprecated** \| **Index** \| **Help** | |  |
| **PREV**   **NEXT** | **FRAMES**    **NO FRAMES**     **All Classes** |  |


---
